# Discovery of an Endosymbiotic Nitrogen-Fixing Cyanobacterium UCYN-A in *Braarudosphaera bigelowii* (Prymnesiophyceae)

**DOI:** 10.1371/journal.pone.0081749

**Published:** 2013-12-04

**Authors:** Kyoko Hagino, Ryo Onuma, Masanobu Kawachi, Takeo Horiguchi

**Affiliations:** 1 Institute for Study of the Earth’s Interior, Okayama University, Yamada, Misasa, Tottori, Japan; 2 Department of Natural History Sciences, Graduate School of Science, Hokkaido University, Kita-ku, Sapporo, Japan; 3 National Institute for Environmental Studies, Onogawa, Tsukuba-City, Ibaraki, Japan; 4 Department of Natural History Sciences, Faculty of Science, Hokkaido University, Kita-ku, Sapporo, Japan; Université Claude Bernard - Lyon 1, France

## Abstract

*Braarudosphaera bigelowii* (Prymnesiophyceae) is a coastal coccolithophore with a long fossil record, extending back to the late Cretaceous (ca. 100 Ma). A recent study revealed close phylogenetic relationships between *B. bigelowii*, *Chrysochromulina parkeae* (Prymnesiophyceae), and a prymnesiophyte that forms a symbiotic association with the nitrogen-fixing cyanobacterium UCYN-A. In order to further examine these relationships, we conducted transmission electron microscopic and molecular phylogenetic studies of *B. bigelowii*. TEM studies showed that, in addition to organelles, such as the nucleus, chloroplasts and mitochondria, *B. bigelowii* contains one or two spheroid bodies with internal lamellae. In the 18S rDNA tree of the Prymnesiophyceae, *C. parkeae* fell within the *B. bigelowii* clade, and was close to *B. bigelowii* Genotype III (99.89% similarity). Plastid 16S rDNA sequences obtained from *B. bigelowii* were close to the unidentified sequences from the oligotrophic SE Pacific Ocean (e.g. HM133411) (99.86% similarity). Bacterial16S rDNA sequences obtained from *B. bigelowii* were identical to the UCYN-A sequence AY621693 from Arabian Sea, and fell in the UCYN-A clade. From these results, we suggest that; 1) *C. parkeae* is the alternate life cycle stage of *B. bigelowii* sensu stricto or that of a sibling species of *B. bigelowii*, and 2) the spheroid body of *B. bigelowii* originated from endosymbiosis of the nitrogen-fixing cyanobacterium UCYN-A.

## Introduction

**Figure 1:**
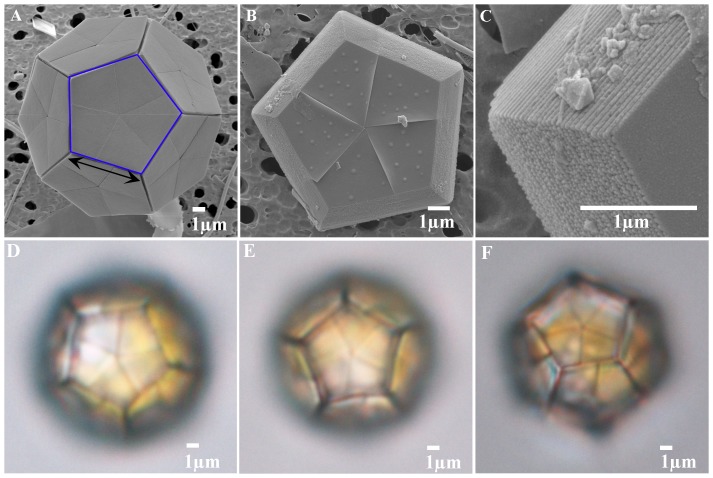
Microscope images of *Braarudosphaera bigelowii*. (A) SEM image of a cell of *B. bigelowii* surrounded by 12 pentaliths (offshore Tomari, 17th June 2012). A pentalith (calcareous scale of the Braarudosphaeraceae) indicated by the blue open pentagon consists of five trapezoidal segments. Black arrow indicates ‘side length of the pentalith’ where the measurements were conducted. (B). SEM image of pentalith of *B. bigelowii* (proximal side) (offshore Tomari, 17th June 2012). (C) Close up of proximal side of a pentalith (Fig. 1B) showing laminar structure. (D) LM image of specimen TMR-scBb-1 (E) LM image of specimen TMR-scBb-7. (F) LM image of specimen TMR-scBb-8.

The family Braarudosphaeraceae are single-celled coastal phytoplanktonic algae characterized by bearing calcareous scales with five-fold symmetry, called pentaliths (Fig. 1A-C). The phylogeny and ecology of the Braarudosphaeraceae are of considerable interest since they have a long fossil record extending back to the early Cretaceous [1], and they are one of the survivors of the K/Pg mass extinction that resulted in the demise of ca. 76% of species with fossil record [2] including ca. 90% of coccolithophores (so called calcareous nannofossils) [1]. In the geological past, the Braarudosphaeraceae contained as many as six genera and many species, but their diversity has declined since the Eocene [3], [4] and only two extant species, *Braarudosphaera bigelowii* (Gran & Braarud) Deflandre and *B. magnei* Lefort, have been described. *B. bigelowii* has a fossil record from the Late Cretaceous (ca. 100 Ma)[1], and its fossils have typically been reported from neritic sediments, although they are occasionally abundant in pelagic sediments, e.g., in lower Paleogene sediments immediately above the K/Pg mass extinction level as well as in the Oligocene Diversity Minimum [5], [6]. *B. magnei* has not been recorded since its first description from seawater from Roscoff, France, and does not have a fossil record [7], [8]. Unlike other members of the Braarudosphaeraceae that form a dodecahedral exotheca with twelve pentaliths [9], [10], *B. magnei* forms a spherical exotheca with many pentaliths [7]. Although *B. magnei* is assigned to *Braarudosphaera*, its phylogenetic affinity has not been verified by either detailed morphological observations or molecular genetics.

Despite numerous attempts, *B. bigelowii* has never been successfully maintained in culture (pers. comm. I. Probert, and authors own experience), and the phylogenetic relationships between the Braarudosphaeraceae and other protists have only recently been determined. Using a single cell PCR technique [11], Takano et al.[12] successfully obtained 18S rDNA sequences of two different-sized cells of *B. bigelowii*. They revealed that *B. bigelowii* has close affinities with the coccolithophore orders Coccolithales and Isochrysidales, and found 16-bp differences including insertions or deletions between the sequences of the two different-sized *B. bigelowii* cells. Subsequently, Hagino et al.[13] studied genetic and morphometric variation of living *B. bigelowii* from the seas surrounding Japan. They revealed that living *B. bigelowii* consists of at least five 18S rDNA genotypes. Geographic distribution of the genotypes of *B. bigelowii* overlapped each other, and the genotypes were correlated with morphotypes distinguished primarily by pentalith size. Their results indicate that living *B. bigelowii* is a complex of multiple discrete species that differ from each other in the size range of pentaliths and in 18S rDNA sequences, although the morphotypes and genotypes of *B. bigelowii* have not yet been raised to species rank.

Although molecular phylogenetic studies showed a close affinity of *B. bigelowii* to coccolithophores, *B. bigelowii* radically differs from other coccolithophores in the structure of its calcareous scales, leading to doubts as to whether it can be considered as a coccolithophore [14]. *B. bigelowii* produces pentaliths consisting of five segments each with a laminar structure (Figs. 1A-C). It is the only known living taxon that produces calcareous scales with a laminar structure, and there is no information available on either its life cycle or on the mechanism of calcification of the laminar pentaliths. In coccolithophores, the morphology of coccoliths is known to be radically different in different life cycle stages [15], [16]. Members of the Coccolithales, Syracosphaerales, and Zygodiscales all produce heterococcoliths formed of a radial array of complex crystal units in their diploid phase, and holococcoliths formed of numerous minute (c.a. 0.1 µm) euhedral crystallites in their haploid phase [8]. Members of the Nöelaerhabdaceae (Isochrysidales) also produce heterococcoliths in their diploid phase, but do not produce calcareous scales in their haploid phase [8], [17]. Coccolith morphology can thus be radically different within a single species depending on ploidy level.

Symbiotic associations between unicellular cyanobacteria and planktonic eukaryotes are widespread phenomena in the oceans and can be found in dinophysoid dinoflagellates, radiolarians and ciliate tintinnids [18], [19]. In these symbioses, the cyanobacteria are thought to provide nitrogen to their hosts [18]. Recently, a new type of symbiotic partnership between cyanobacteria and prymnesiophyte algae has been reported [20]. Thompson et al. [20] reported close phylogenetic relationships between *B. bigelowii*, *Chrysochromulina parkeae* (Prymnesiophyceae), and a prymnesiophyte that forms an extracellular symbiotic association with a cyanobacterium. In order to further investigate these intriguing results, we conducted transmission electron microscopic and molecular phylogenetic studies of *B. bigelowii*. In addition, during TEM observations we discovered the presence within *B. bigelowii* cells of one or two characteristic spherical structures which resemble the ‘spheroid body’ of the freshwater diatom *Rhopalodia gibba* (Rhopalodiales) [21]. Because the latter structure was found to be an endosymbiont of cyanobacterial origin based on 16S rDNA sequences [22], we also sequenced this gene from *B. bigelowii*.

## Materials and Methods

We do not involve human, animal, vertebrate samples in this study. Seawater and plankton samples used in this study were collected in public ports and offshore, in areas of open public access. No specific permissions were required for sampling of seawater (including microplankton) in the localities. We confirmed that the species that we used in this study are not endangered or protected species.

### Electron microscopy studies of *Braarudosphaera*

For TEM observations of *B. bigelowii*, samples containing living cells of *B. bigelowii* were selected from natural seawater samples collected for regular floristic studies of coccolithophores (Hagino, unpublished). Seawater samples collected from sea surface level (0m) of Tosa Bay, Kochi, Japan (33°38’N 133°54’E) on 1st June 2007 and from sea surface level (0m) of Tomari Port, Tottori, Japan (35°33’E 133°55’E) on 18th June 2011 by a bucket were used for TEM preparation. Samples (10 liters) were first pre-filtered through a 50 µm mesh plankton net (Sefar Inc. DIN-110) in order to remove large plankton. Pre-filtered samples were concentrated using a piece of 1 µm mesh-size plankton net (Sefar Inc. NY1-HD) placed on a kitchen sieve, then the concentrated seawater was centrifuged at 1,000 rpm for 3 min. The pellet was fixed for 3 hours at room temperature with 0.5 ml of 3.5% glutaraldehyde made up in sterile seawater, and subsequently rinsed in 0.1 M sterile mineral water (pH 8.0) three times. The sample was then post-fixed for 2 h in 2% OsO_4_ made up in mineral water (pH 8.0). The sample was dehydrated through an acetone series and finally embedded in resin (Agar Low Viscosity Resin, Agar Scientific Limited, England). A small amount of resin containing *Braarudosphaera* cells and other plankton was transferred onto a piece of transparency film and the resin was sandwiched by another transparency film. This was polymerized in an oven and regions of the resin containing *Braarudosphaera* cells were then selected under a microscope for sectioning. Sections were cut using a diamond knife on an ultramicrotome (LEICA EM UC6, Germany) and placed on formvar-coated grids. Observations were made using a HITACHI H-7650 transmission electron microscope at Hokkaido University, Japan.

### Phylogenetic analyses of 18S rRNA, 16S rRNA, and *nif*H genes

Seawater samples collected from surface level (0m) of Tomari Port by a bucket on 22nd November 2012 and on 18th June 2013 were selected for molecular phylogenetic studies of *B. bigelowii* from the samples originally collected for regular floristic studies of coccolithophores in the area (Hagino, unpublished). 10 liters of seawater was pre-filtered through a 50-µm mesh-size plankton net (Sefar Inc. DIN-110) and then concentrated using a piece of 1-µm mesh-size plankton net (Sefar Inc. NY1-HD) placed on a kitchen sieve. A living cell of *B. bigelowii* labeled as TMR-scBb-1 was isolated from the sample collected on 22nd November 2012, and the cells labeled as TMR-scBb-7 and -8 were isolated from the sample collected on 18th June 2013 using a micropipette under an inverted light microscope (Olympus CKX41). Each cell was carefully cleaned with sterile seawater using a micropipette under the inverted light microscope. Each cell was observed (magnification x1500) and photographed using a Nikon E600POL microscope (Figs. 1D-F), and then subjected to single cell PCR amplification as outlined in Takano and Horiguchi [11].

In the beginning of this study, we used 18S and 16S rDNA primers published by previous studies for PCR amplification and sequencing (Table 1). Based on the 16S rDNA sequences obtained from the experiments, we designed new primers for bacterial and plastid16S rRNA genes of *B. bigelowii* and then conducted subsequent PCR amplification of 16S rDNA using published and our new primers. Details of the experiments are described below.

**Table 1:** Oligonucleotide primers used for amplification and sequencing.

Code	SD	Sequence	References
SR1	F	TACCTGGTTGATCCTGCCAG	[49]
SR1c	F	CAGTAGTCATATGCTTGYCTC	[49]
SR2TAK	F	CATTCAAATTTCTGMCCTATC	[11]
SR3	R	AGGCTCCCTGTCCGGAATC	[49]
SR4	F	AGGGCAAGTCTGGTGCCAG	[49]
SR5TAK	R	ACTACGAGCTTTTTAACYGC	[11]
SR7TAK	R	TCCWTGGCAAATGCTTTCGC	[11]
SR8TAK	F	GGATTGACAGATTGAKAGCT	[11]
SR9	R	AACTAAGAACGGCCATGCAC	[49]
SR12	R	CCTTCCGCAGGTTCACCTAC	[49]
27F	F	AGAGTTTGATCMTGGCTCAG	[50]
1492R	R	GGYTACCTTGTTACGACTT	[47]
CYA359F	F	GGGGAATYTTCCGCAATGGG	[51]
Nitro 821R	R	CAAGCCACACCTAGTTTC	[52]
16SBbsp726F	F	GCAACTGACACTCAGGGA	This study
Bbplastid 445F	F	AGAAAGAAGTTCTGACGGTACTTG	This study
Bbplastid 604F	F	TAGGAAAGCGATGGAAACTGATAG	This study
Bbplastid 628R	R	CTATCAGTTTCCATCGCTTTCCTA	This study
Bbplastid 830R	R	CTGYMCAASTTGCACAACAYCTAGTA	This study
nif H1	F	TGYGAYCCNAARGCNGA	[53]
nif H2	R	ADNGCCATCATYTCNCC	[53]
nif H3	F	ATRTTRTTNGCNGCRTA	[53]
nif H4	R	TTYTAYGGNAARGGNGG	[53]

The first reaction of PCR of the specimen TMR-scBb-1 was performed using external primers for 18S rDNA (SR1 and SR12) and for 16S rRNA primers (27F and 1492R). The first reaction of PCR of the specimens TMR-scBb-7 and -8 were performed using external primers for 18S rDNA (SR1 and SR12), for 16S rRNA primers (27F and 1492R), and nitrogenase primers (nifH4 and nifH3) (Table 1). The conditions for the first PCR round for the specimen TMR-scBb-1 were one initial denaturation step at 98°C for 60s, followed by 35 cycles of denaturation at 94°C, annealing at 50°C for 30s and extension at 72°C for 60s. The conditions for the first PCR reaction for the specimens TMR-scBb-7 and -8 were one initial denaturation step at 98°C for 60s, followed by 35 cycles of denaturation at 94°C, annealing at 47°C for 30s and extension at 72°C for 60s.

In the second PCR reaction in the beginning of the experiments, short internal sequences of 18S and 16S rDNA genes were amplified using 0.5 µL of the first PCR product of the specimen TMR-scBb-1 as the DNA template with three pairs of 18S rDNA primers (SR1c-SR5tak, SR4-SR9, SR8-SR12) and three pairs of 16S rDNA primers (27F-Nitro821R and CYA359F-Nitro821R, and CYA 359F-1492R) (Table 1). In order to check possible occurrence of contamination, negative control samples without template were subjected to PCR amplification together with the samples containing template. The PCR conditions for the second reaction were one initial denaturation step at 98°C for 30s, followed by 10 cycles of denaturation at 96°C for 30s, annealing at 57°C for 30s and extension at 72°C for 30s, 10 cycles of denaturation at 96°C for 30s, annealing at 56°C for 30s and extension at 72°C for 30s, and 10 cycles of denaturation at 95°C for 30s, annealing at 55°C for 30s and extension at 72°C for 30s. The temperature profile was completed by a final extension cycle at 72°C for 4 min. In order to determine the amplification efficiency and possible occurrence of contamination, 2.5 µl of each second round PCR products were visualized by agarose electrophoresis. The successfully amplified products of the second round of PCR amplifications without contamination were sequenced directly using the ABI PRISM BigDye Terminator Cycle Sequencing Kit ver. 1.1 (Perkin-Elmer, Foster City, CA, USA) on a DNA auto sequencer ABI PRISM 3130 Genetic Analyzer (Perkin-Elmer) in the Faculty of Science, Hokkaido University, Japan. The results were confirmed by sequencing both forward and reverse strands.

The 16S rRNA gene sequences obtained from the specimen TMR-scBb-1 in the experiments described above were subjected to a Blast Search. Sequences obtained by primer pairs of 27F-Nitro821R and by primer pairs of CYA359F-Nitro821 were identical to each other, and close to the bacterial16S rDNA sequences of uncultured nitrogen-fixing cyanobacterium UCYN-A. On the other hand, the sequences obtained by the primers CYA359F-1492R were close to the plastid 16S rDNA sequences of members in the class Prymnesiophyceae, Division Haptophyta. Based on the sequences obtained by the ‘CYA359F-Nitro821R’ and by ‘CYA359F-1492R’, we designed one primer for bacterial 16S RNA gene of *B. bigelowii* (16SBbsp726F) and four primers for plastid 16S rRNA gene of *B. bigelowii* (Bbplastid 445F, Bbplastid 604F, Bbplastid 628R, and Bbplastid 830R), respectively (Table 1).

In the subsequent second PCR reactions for the specimen TMR-scBb-1, bacterial 16S and plastid 16S rRNA genes were amplified with three pairs of bacterial 16S rDNA primers (27F-Nitro821R, CYA359F-Nitro821R, and 16SBbsp726F-1492R), and three pairs of plastid 16S rDNA primers (27F-Bbplastid682R, Bbplastid 445F-Bbplastid830R, and Bbplastid604F-1492R), respectively. In the second PCR reactions for the specimens TMR-scBb-7 and -8, four pairs of 18S rDNA primers (SR1c-SR3, SR2tak-SR7, SR4-SR9, SR8-SR12), three pairs of bacterial 16S rDNA primers (27F-Nitro821R, CYA359F-Nitro821R, and 16SBbsp726F-1492R), three pairs of plastid 16S rDNA primers (27F-Bbplastid682R, Bbplastid 445F-Bbplastid830R, and Bbplastid604F-1492R), and one pair of nifH primers (nifH1-nifH2) were used. In all the second PCR reactions, negative control samples without template were subjected to PCR amplification together with the samples with template in order to check possible contamination. The PCR conditions for the second reaction were one initial denaturation step at 98°C for 30s, followed by 30 cycles of denaturation at 96°C for 30s, annealing at 55°C for 30s and extension at 72°C for 30s. The temperature profile was completed by a final extension cycle at 72°C for 4 min. In order to determine the amplification efficiency and possibility of contamination, 2.5 µl of each second round PCR products were visualized by agarose electrophoresis. Agarose electrophoresis suggested that fragments of 18S, plastid 16S, and bacterial 16S rRNA genes were successfully amplified without contamination, however, the *nif*H gene was not amplified for an unknown reason.

The products of the second reaction of PCR amplifications of the 18S and 16S rRNA genes were sequenced directly using the ABI PRISM BigDye Terminator Cycle Sequencing Kit ver. 1.1 (Perkin-Elmer, Foster City, CA, USA) on a DNA auto sequencer Applied Biosystems 3130xl Genetic Analyzer in the Institute of Plant Science and Resources, Okayama University, Japan. The results were confirmed by sequencing both forward and reverse strands. Three 18S rDNA sequences (AB778293, AB847980 and AB847981), three plastid 16S rDNA sequences (AB847984, AB847985 and AB847986) and three bacterial 16S rDNA sequences (AB778292, AB847982 and AB847983) obtained in this study were deposited into the DNA Data Bank of Japan (http://www.ddbj.nig.ac.jp/index-e.html).

For the phylogenetic analysis of the 18S rRNA gene, a total of 44 OTUs including two members of the Pavlovophyceae as an out-group, were obtained from GenBank. The 18S rDNA sequence of *C. parkeae* strain Kawachi (AM490994), which was published in Medlin et al. [23], was originally obtained from a culture strain of *C. parkeae* established by M. Kawachi (Kawachi, pers. comm.). Since 18S rDNA sequences of *B. bigelowii* from the specimens TMR-sc-Bb-1 (AB778293), TMR-sc-Bb-7 (AB847980), and TMR-sc-Bb-8 (AB847981) obtained in this study were identical to the previously reported six sequences of *B. bigelowii* Genotype III (AB250784, AB847974-AB847978), we used the previously published sequence AB250784 as a representative of the Genotype III in this analysis. Taxonomic classification of the Haptophyta followed Edvardsen et al. [24]. For the phylogenetic analysis of the plastid 16S rRNA gene, a total of 48 OTUs including a member of the Pavlovophyceae as an out-group were obtained from GenBank. Since the plastid 16S rDNA sequences of *B. bigelowii* from the specimens TMR-sc-Bb-1 (AB847984), TMR-sc-Bb-7 (AB847985), and TMR-sc-Bb-8 (AB847986) obtained in this study were identical to the each other, we only included the sequence from TMR-scBb-1 (AB847984) as a representative of the plastid 16S rDNA of *B. bigelowii* in this analysis. For the phylogenetic analysis of the bacterial 16S rRNA gene, a total of 33 OTUs, including *Gloeobacter violaceus* (Gloeobacterales) as an out-group, were used. Since the bacterial 16S rDNA sequences of *B. bigelowii* from the specimens TMR-sc-Bb-1 (AB778292), TMR-sc-Bb-7 (AB847982), and TMR-sc-Bb-8 (AB847983) were identical to the each other, we only included the sequence from TMR-scBb-7 (AB847982) as a representative of the bacterial 16S rDNA of *B. bigelowii* in this analysis. Each set of sequences was aligned using Clustal W (http://www.genome.jp/tools/clustalw/).

For the 18S, bacterial 16S and plastid 16S rRNA genes, phylogenetic trees were constructed based on Maximum Likelihood (ML), Neighbor Joining (NJ), and Maximum Parsimony (MP) methods using PAUP version 4.0b10 [25], and also based on Bayesian inference (BI) using Mr. BAYES v3.1.2 [26]. The use of Neighbor Joining and Maximum Parsimony analyses provided similar results (not shown) to those of ML analyses, therefore we describe only the method and results of ML and BI analyses in this study. Substitution models were selected using Modeltest 3.7 [27] and MrModeltest 2.2 [28] for ML and BI, respectively. ML was performed using the heuristic search option with a branch-swapping algorithm Tree bisection-reconnection (TBR). For the ML analyses, the NJ tree was used as a starting tree. Bootstrap analyses with 1000 replicates for ML analyses were applied to examine the robustness and statistical reliability of the topologies [29]. Markov chain Monte Carlo iterations for the BI analysis of 18S, plastid 16S and bacterial 16S rRNA genes were carried until the average standard deviations of split frequencies below 0.01, indicated convergence of the iterations.

For ML analysis of the 18S rRNA gene, a likelihood score (-ln*L*  =  9116.6914) was obtained under the TrN+I+G model with the following parameters: assumed nucleotide frequencies A =  0.2400, C =  0.2192, G =  0.2799, and T =  0.2608; substitution-rate AC =  1, AG =  1.4310, AT =  1, CG =  1, CT =  4.0184, GT =  1; proportion of sites assumed to be invariable  =  0.5941; rates for variable sites assumed to follow a gamma distribution with shape parameter  =  0.5669, and number of rate categories  =  4, estimated by Modeltest 3.7. For the BI analysis of the 18S rRNA gene, GTR+I+G model was selected by the MrModeltest, and the Markov chain Monte Carlo iterations were carried out until 1.5 million generations.

For the ML analysis of plastid 16S rRNA gene, a likelihood score (-ln*L*  =  4482.6802) was obtained under the GTR+I+G model with the following parameters: assumed nucleotide frequencies A =  0.2632, C =  0.1814, G =  0.3033, and T =  0.2520; substitution-rate AC =  0.96991, AG =  7.9722, AT =  2.4899, CG =  1.7909, CT =  11.6406, GT =  1.0000; proportion of sites assumed to be invariable  =  0.3964; rates for variable sites assumed to follow a gamma distribution with shape parameter  =  0.4353, and number of rate categories  =  4, estimated by Modeltest 3.7. For the BI analysis of the plastid 16S rRNA gene, GTR+I+G model was selected by the MrModeltest, and the Markov chain Monte Carlo iterations were carried out until 16.5 million generations.

For the ML analysis of the bacterial 16S rRNA gene, a likelihood score (-ln*L*  =  6723.6357) was obtained under the TrN+I+G model with the following parameters: assumed nucleotide frequencies A =  0.2575, C =  0.2219, G =  0.3085, and T =  0.2120; substitution-rate AC =  1, AG =  3.0334, AT =  1, CG =  1, CT =  4.1247, GT =  1; proportion of sites assumed to be invariable  =  0.6197; rates for variable sites assumed to follow a gamma distribution with shape parameter  =  0.6451, and number of rate categories  =  4, estimated by Modeltest 3.7. For the BI analysis of the bacterial 16S rRNA gene, GTR+I+G model was selected by MrModeltest, and the Markov chain Monte Carlo iterations were carried out until 1.5 million generations.

## Results

### Morphological studies

**Figure 2:**
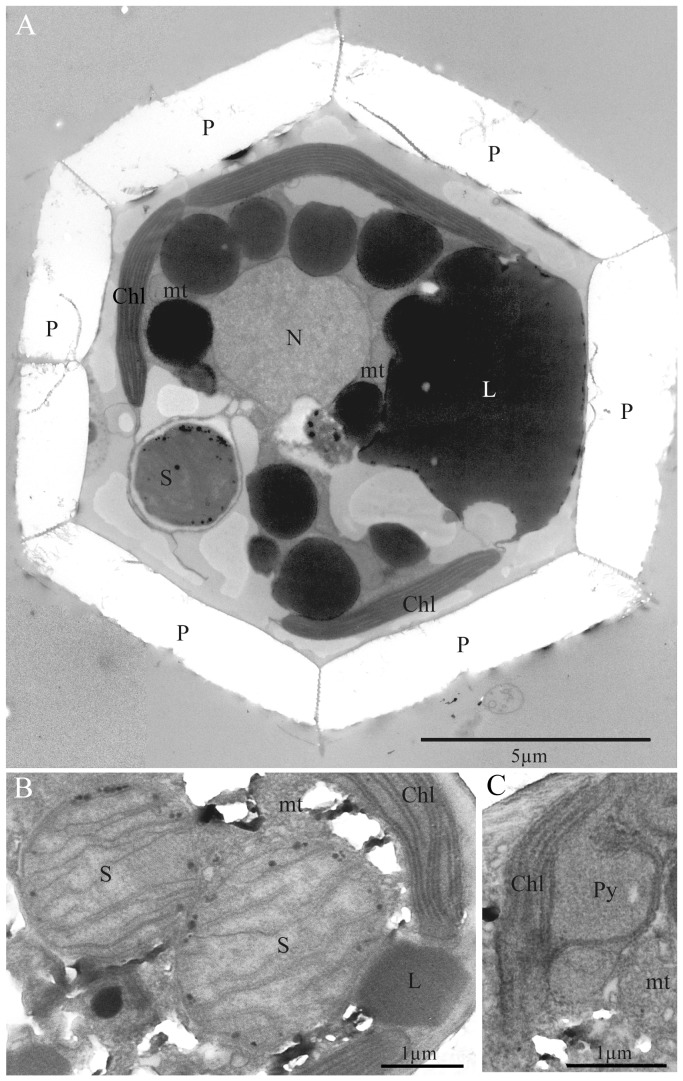
TEM images of *Braarudosphaera bigelowii* specimens -A and -B. (A) *B. bigelowii* specimen-B from offshore Tomari port, Tottori, showing nucleus (N), chloroplasts (Chl), lipid globules (L), pentaliths (P), mitochondria (mt) and spheroid body (S). (B) *B. bigelowii* specimen -A from Tosa Bay, Kochi, Japan, showing detail of spheroid bodies (S). Note that the structure contains about 10 lamellae. The chloroplast (Chl) and lipid globules (L) can also be seen. (C) Detail of chloroplast of *B. bigelowii* specimen -A from Tosa Bay, Kochi, Japan, showing a bulging type of pyrenoid (Py). The mitochondrial profile (mt) can be seen.

**Figure 3:**
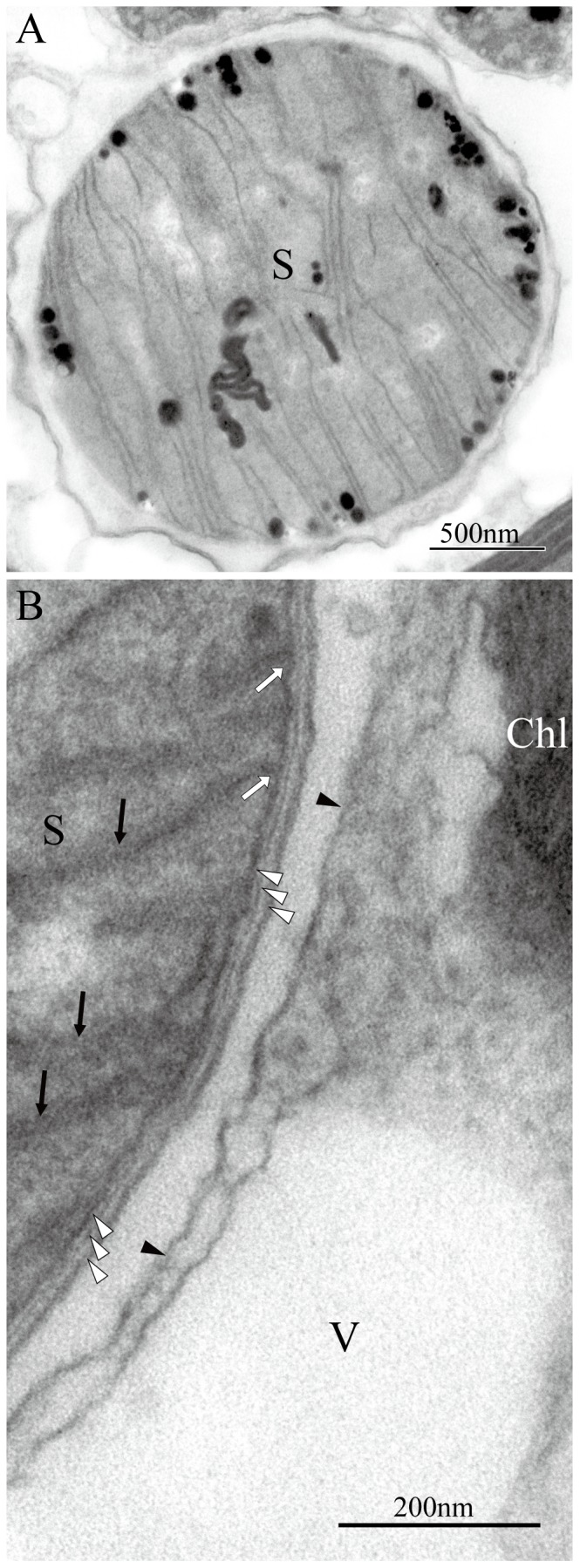
TEM images of spheroid body (S) in *Braarudosphaera bigelowii* specimen-C from offshore Tomari port. (A) Spheroid body in *B. bigelowii* specimen-C. (B) Detail of spheroid body of Figure 3 (a), showing internal lamellae (arrow). Note that the spheroid body is separated from the host by a single membrane (arrowhead) and the envelope of the spheroid body itself consists of three layers (open arrowhead), from outside to inward, possibly corresponding to outer membrane, peptidoglycan wall and plasma membrane of gram-negative bacteria. Membranes of lamellae are attached to plasma membrane (open arrow).Vacuole (V) is observed around the spheroid body.

The three specimens of *B. bigelowii* observed under a TEM were almost the same size, i.e. approximately 15 µm in cell diameter. From the cell diameter, the side length of the pentaliths of these specimens was estimated to be ca. 5.6 µm. These specimens therefore correspond in size to *B. bigelowii* Intermediate form-B of Hagino et al.[13]. Fig. 2A shows the general arrangement of the organelles in *B. bigelowii*. The cell is surrounded by thick pentaliths, the cytoplasm contains a spherical nucleus, chloroplasts, mitochondria with tubular cristae, and large lipid globules, which occupy a substantial part of the cytoplasm (Fig. 2A). The chloroplasts are thin, plate-like, and are located in the periphery of the cell. They do not have girdle thylakoids, but the possession of a bulging pyrenoid has been confirmed (Fig. 2C). In addition to these ordinary organelles and storage products, the cell always contains one or two spherical structures (Fig. 2A-B, 3A-B). These structures are almost spherical and approximately 2 µm in diameter. They are separated from the host by a single membrane (Fig. 3B). The outside of the spherical structure is well-vacuolated, and the vacuole membrane is often located close to the single membrane separating the spherical structure from the host, as a result, the spherical structure often appears to be surrounded by a double membrane (Fig. 3A-B). The connection of these membranes to endoplasmic reticulum (outer nuclear envelope) was not detected in this observation. The envelope of these spherical structures consists of three layers; outer membrane, middle thin membrane-like layer and inner cytoplasmic membrane (Fig. 3B). We believe that these layers correspond to, from outside to inward, outer membrane, peptidoglycan wall and plasma membrane of gram-negative bacteria. Their matrices are traversed by many (up to 30) lamellae (flattened vesicles) (Figs. 2A-B, 3A-B). The length of these lamellae varied and each structure is attached to the inner membrane with its proximal end (Fig. 3B). These spherical structures also contain small osmiophilic globuli in their matrices (Figs. 2A-B, 3A-B).

### Classification of *B. bigelowii* morphotype used for the molecular studies

The size of pentaliths of specimen TMR-scBb-1, -7, and -8 measured on a light microscope images were ca. 5.5, 5.6, 5.4 µm in side length, respectively (Fig. 2). Size of these three specimens are included in the size range of the Intermediate form-B (5.3-7.0 µm), which was considered to be *B. bigelowii* sensu stricto by Hagino et al. [13].

### Molecular phylogenetic study based on 18S rDNA sequences

18S rDNA sequences were determined from three specimens TMR-scBb-1 (1739-bp, AB778293), TMR-scBb-7 (1739-bp, AB847980), and TMR-scBb-8 (1739-bp, AB847981). These sequences were identical to each other, and to that of *B. bigelowii* Genotype III (AB250784) which was obtained from specimens of Intermediate form-B [13]. The 18S rDNA sequence of *C. parkeae* (AM490994) was closest to the sequence of *B. bigelowii* Genotype III (99.89% similarity).

**Figure 4:**
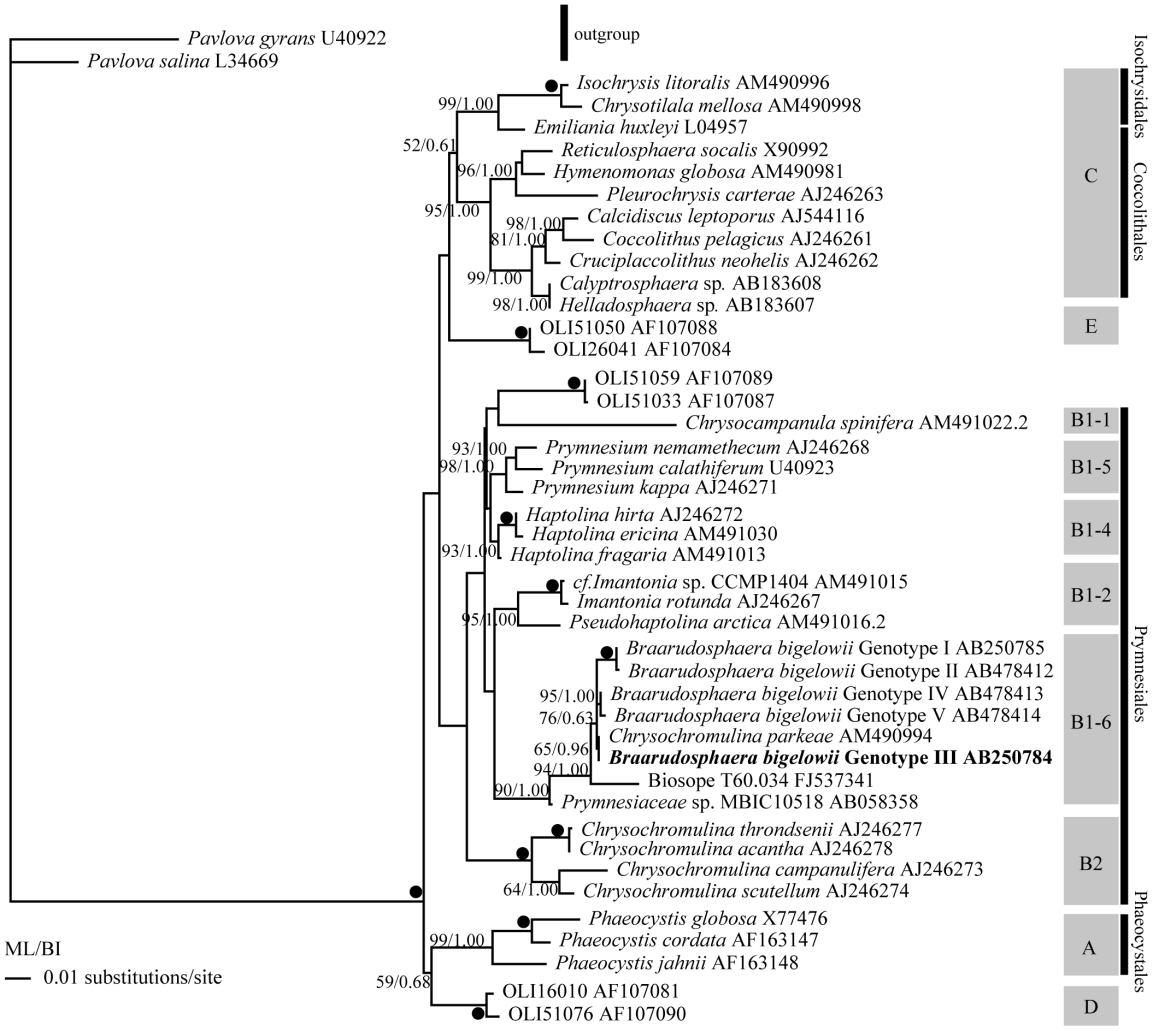
Phylogenetic tree based on 18S rDNA sequences using the Maximum Likelihood method. Representatives of the Pavlovophyceae were used as an out-group. Asterisks refer to the clade names of Edvardsen et al. [24]. The numbers on each node indicate the bootstrap values from ML analysis and posterior probability of BI analysis. Solid circles indicate the clades supported by very high bootstrap values (100%) and posterior probability (1.00) by all analyses (ML, NJ, MP, and BI).

The topologies of phylogenetic trees obtained by ML and BI analyses were similar to each other, and similar to the tree obtained by Edvardsen et al. [24]. Here we show only the ML tree with bootstrap consensus values obtained from ML and posterior probabilities obtained from BI analysis (Fig. 4). The phylogenetic position of the clade *B. bigelowii* + *C. parkeae* in our trees differs from those in the phylogenetic trees of Takano et al.[12] and Hagino et al.[13], but are similar to those in the trees of Edvardsen et al.[24] and Thompson et al.[20]. The five *B. bigelowii* genotypes and *C. parkeae* made a clade with moderate bootstrap support and low posterior probability (76/0.63; ML/BI). The *B. bigelowii* + *C. parkeae* clade clustered with the Biosope T60.64 from SE Pacific Ocean [20], [30] with high boot strap support and posterior probability (94/1.00; ML/BI), and in turn this clade clustered with *Prymnesiaceae* sp. MBIC10518 with high bootstrap support and posterior probability (90/1.00; ML/BI). Due to low bootstrap support and posterior probability, the phylogenetic relationships of the ‘*B. bigelowii* + *C. parkeae* + Biosope T60.64 and *Prymnesioceae sp.* MBIC10518’ clade with other Prymnesisales (*Imantionia* spp., *Prymnesium* spp., and *Haptolina* spp.) was uncertain.

### Molecular phylogenetic study based on plastid 16S rDNA sequences

**Figure 5:**
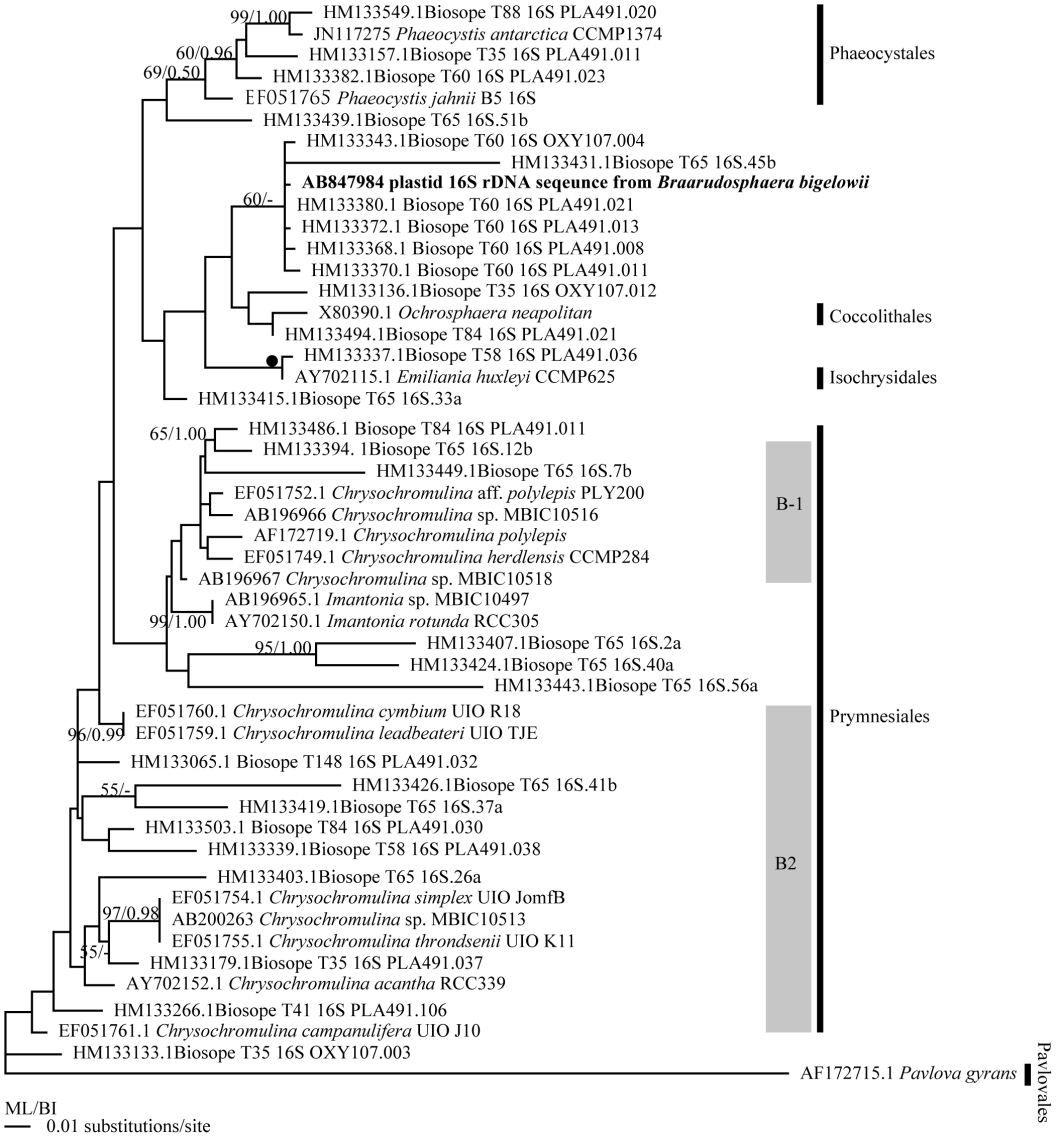
Phylogenetic tree based on plastid 16S rDNA sequences using the Maximum Likelihood method. Representatives of the Pavlovophyceae were used as an out-group. The numbers on each node indicate the bootstrap values from ML analysis and posterior probability of BI analysis. Clade names B-1 and B-2 refer the clade names of Shi et al.[31] Solid circles indicate the clades supported by very high bootstrap values (100%) and posterior probability (1.00) by all analyses (ML, NJ, MP, and BI).

Plastid 16S rDNA sequences were determined from three specimens TMR-scBb-1 (1375-bp, AB847984), TMR-scBb-7 (1376-bp, AB847984), and TMR-scBb-8 (1375-bp, AB847984). The three sequences obtained were identical to each other (100% identity), and close to the sequences from oligotrophic SE Pacific samples T60 and T65 (HM133411, HM133414, HM133418, and HM133377) of Shi et al.[31] (99.86% similarity) in the region in which they overlapped (704-bp). The topologies of phylogenetic trees obtained by ML and BI analysis were similar to each other. Here we show only the ML tree with bootstrap consensus values obtained from ML and posterior probabilities obtained from BI analysis (Fig. 5). In the MLand BI trees, the plastid 16S rDNA sequence from *B. bigelowii* made a clade with the sequences from Biosope T60 and T65 (HM133331, HM133343, HM133368, HM133370, HM133372, and HM13330) with low bootstrap support and without posterior probability (60/-; ML/BI). The clade containing the plastid 16S rDNA of *B. bigelowii* clustered with the sequences from coccolithophores (*Ochrosphaera neapolitana* and *Emiliania huxleyi*) without bootstrap support and posterior probability. Due to low bootstrap supports and posterior probability, the relationships between the plastid 16S rDNA sequences examined here are uncertain.

### Molecular phylogenetic study based on bacterial 16S rDNA sequences

Bacterial 16S rDNA sequences were determined from three specimens TMR-scBb-1 (1330-bp, AB778292), TMR-scBb-7 (1393-bp, AB847982), and TMR-scBb-8 (1390-bp, AB847983). The three sequences obtained were identical to each other (100% identity), and the same as the uncultured cyanobacterium sequence AY621693 from the Arabian Sea in the overlapping sites (440-bp).

**Figure 6:**
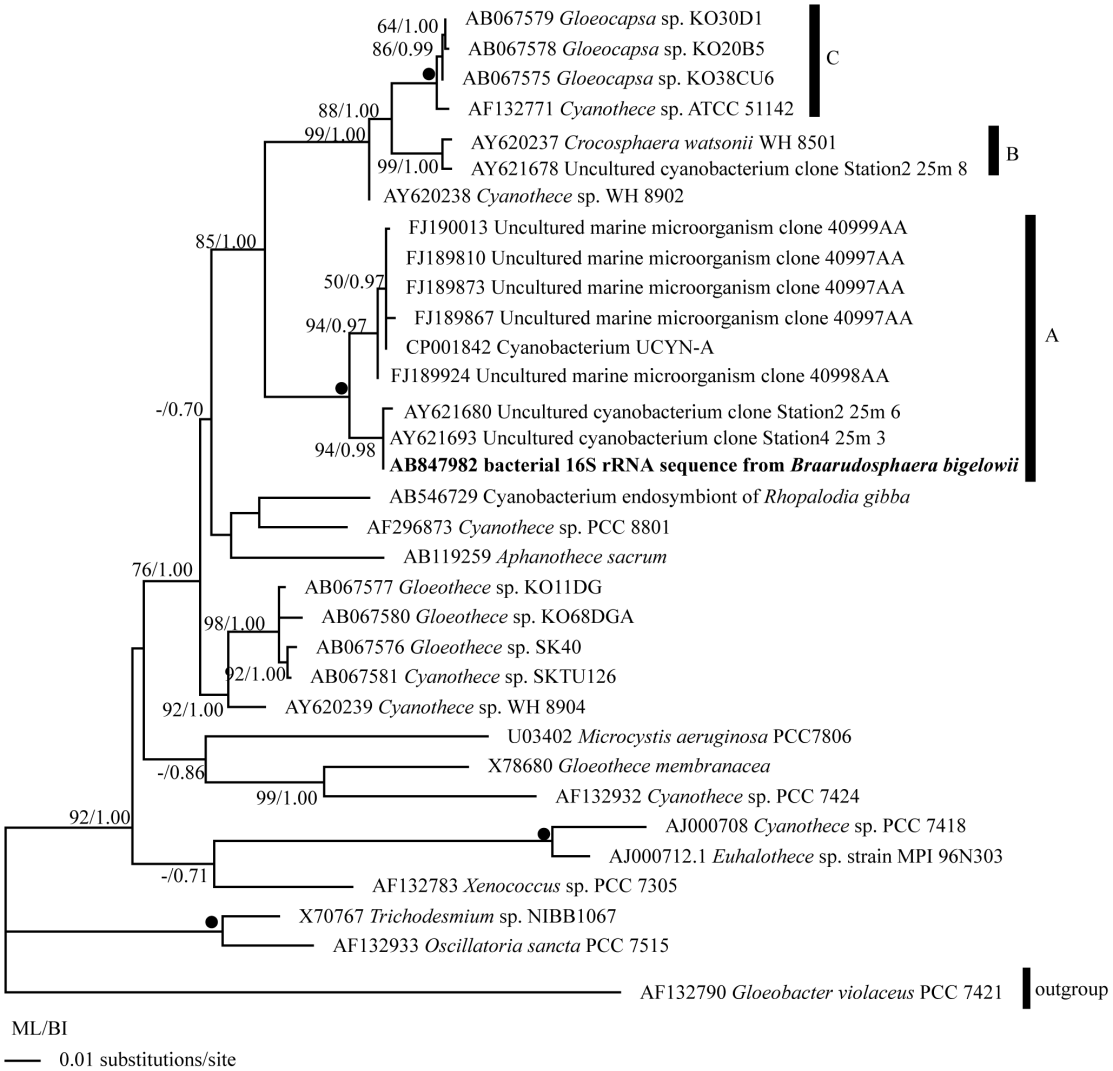
Phylogenetic tree based on 16S rDNA sequences using the Maximum Likelihood method. *Gloeobacter violaceus* was used as an out-group. The numbers on each node indicate the bootstrap values from ML analysis and posterior probability of BI analysis. Clade names A, B, and C refer Taniuchi et al.[32] Solid circles indicate the clades supported by very high bootstrap values (100%) and posterior probability (1.00) by all analyses (ML, NJ, MP, and BI).

The topologies of phylogenetic trees obtained by ML and BI analysis were similar to each other, and similar to the tree shown in Taniuchi et al.[32]. Here we show only the ML tree with bootstrap consensus values obtained from ML and posterior probabilities obtained from BI analysis (Fig. 6). In all trees examined, the bacterial 16S rDNA sequence from *B. bigelowii* was included in the clade of UCYN-A with very high bootstrap supports and posterior probability (100/1.00; ML/BI). Due to low bootstrap supports and posterior probability, the phylogenetic relationships of the UCYN-A clade with other cyanobacterial groups were uncertain. In the UCYN-A clade, the 16S rDNA sequences from *B. bigelowii* formed a sub-clade with the two UCYN-A sequences from the Arabian Sea (AY621680 and AY621693) with high bootstrap supports and posterior probability (94/0.98; ML/BI), separate from the other UCYN-A sequences from the ALOHA station, offshore Hawaii.

## Discussion

Our electron microscope studies show that *B. bigelowii* cells contain one or two spherical structures in addition to standard prymnesiophyte organelles (Figs 2-3). The spherical structures of *B. bigelowii* resemble the ‘spheroid body’ of the freshwater diatom *Rhopalodia gibba* [21], which has been demonstrated to be a nitrogen-fixing apparatus of cyanobacterial origin [22]. The spheroid body of *R. gibba* is 4 to 6 µm in width, 5 to 7 µm in length and was described as possessing a five-layered wall [21], while the spherical structure of *B. bigelowii* is 2 µm in diameter and has a three layered wall. However, careful examination of Figs. 5 and 6 of Drum and Pankratz [21] revealed that their five layer includes two internal layers between outer membrane – peptidoglycan and peptidoglycan – plasma membrane, respectively. Therefore, the cell wall of both structures have essentially the same construction, outer membrane, peptidoglycan wall and plasma membrane, i.e. typical cell wall of gram-negative bacteria, which include cyanobacteria. However, Prechtl et al.[22] described the spheroid body in *R. gibba* as being surrounded by only two membranes and no wall structure was observed. At this moment, we do not know the cause of discrepancies between the results of Drum and Pankratz [21] and Prechtl et al.[22] even though they observed endosymbionts of the same diatom species. In addition, the spherical structure of *B. bigelowii* shares similar features with the spheroid body of *R. gibba* with regard to arrangement of internal lamellae [21] and separating membrane from the host. Therefore, the two structures have essentially the same structure except for their cell sizes and based on the structural similarity, we call the spherical structure of *B. bigelowii* the “spheroid body” hereafter (Figs. 2-3). Although *B. bigelowii* apparently belong to the Prymnesiales (Fig. 4), this type of spheroid body has never previously been reported from other members of the order [33], [34], [35], [36], [37].

Molecular phylogenetic analysis based on 18S rDNA sequences reveal that *C. parkeae* is included in the *B. bigelowii* clade with moderate to high bootstrap values and high posterior probability and is very closely related to *B. bigelowii* Genotype III (99.89% similarity) (Intermediate form-B) (Fig. 4). *C. parkeae* is an elongate motile unicellular alga with two flagella, a haptonema, and characteristic organic scales [38]. It does not produce calcareous scales and is very different from *B. bigelowii* in almost all respects, so it was surprising to find that *C. parkeae* was included in the *B. bigelowii* clade.

Despite the radically different cell and scale morphologies, *C. parkeae* and *B. bigelowii* genotype III, which correspond to *B. bigelowii* sensu stricto [13], have almost identical 18S rDNA sequences (99.89% similarity). *B. bigelowii* is a species complex consisting of multiple discrete species which can be separated from each other based on size of pentalith and on 18S rDNA sequences [13]. These results suggest that *C. parkeae* is an alternate life cycle stage of *B. bigelowii* sensu stricto or that of a sibling species of *B. bigelowii*. Numerous coccolithophores are known to produce different types of coccoliths during the diploid and hapoid life cycle stages [8], [16]. In *Emiliania huxleyi*, the non-motile, coccolith-bearing diploid phase alternates with a non-calcified (but organic scale bearing) haploid motile phase in a haplo-diplontic life cycle [39]. By analogy, we suggest that *C. parkeae* is an alternate life cycle form of *B. bigelowii.* Taxonomically, the name *B. bigelowii* (Gran & Braarud) Deflandre [40] has priority over *C. parkeae* Green & Leadbeater [38]. Although it might be assumed by analogy with coccolithophores such as *E. huxleyi* that the calcifying (*B. bigelowii*) phase is diploid and the non-calcifying (*C. parkeae*) phase is haploid, we cannot confirm this hypothesis since the culture strain of ‘*C. parkeae*’ has been lost (Kawachi, pers. comm.) and *B. bigelowii* has never been cultured. In order to clarify the life cycle of *B. bigelowii*, culture strains of both *B. bigelowii* and ‘*C. parkeae*’ need to be (re)established.

The plastid 16S rDNA sequences (1375-1376-bp) obtained from three *B. bigelowii* isolates were identical to each other, and were close to that of uncultured prymnesiophyte sequences from oligotrophic SE Pacific open samples T60 and T65 (HM133411, HM133414, HM133418, and HM133377) (99.86% similarity) [31] in the overlapping sites (704bp). It was an unexpected result since *B. bigelowii* have been thought to be a coastal-neritic species. Living *B. bigelowii* usually occurs in coastal-neritic waters [41], [42]. Tanaka [43] studied thanatocoenosis of coccolithophores in surface sediments in the seas surrounding Japan. He showed that the distribution of remains of *B. bigelowii* is essentially restricted to neritic surface sediments shallower than 70m. The affinity of *B. bigelowii* with shallow coastal water has also been reported from the Persian Gulf [44] and the Bay of Sendai [45]. Fossil pentaliths of the *B. bigelowii* is easily distinguishable from the marine sediment under a cross-polarized light microscope, they are hardly overlooked in the micropaleontological studies. Lack of report of fossils of the Braarudosphaeraceae from the enormous number of micropaleontological studies of the open ocean sediments except for the studies of the sediments of the specific time interval (early Danian immediately after the K/Pg event and in the Oligocene Diversity Minimum) [6], [46] also supports that distribution of *B. bigelowii* is usually restricted in neritic waters, and occurrence of *B. bigelowii* in the modern open ocean is very exceptional. The close affinity of the plastid 16S rDNA of *B. bigelowii* (this study) to the plastid 16S rDNA from oligotrophic SE Pacific[31] and lack of reports of remains of *B. bigelowii* from open ocean sediments suggests that an unknown species closely related to *B. bigelowii* distributes in open ocean, and that this species does not produce calcareous pentaliths.

The bacterial 16S rDNA sequences obtained from three *B. bigelowii* isolates (1330-1339 bp) were identical to each other, and the same as the sequence AY621693 from the Arabian Sea in the overlapping sites (440bp), and fell in the UCYN-A clade with very high support values (100/1.00; ML/BI) (Fig. 6). As we described in the methods, each isolate of *B. bigelowii* was cleaned with sterile seawater using a micropipette under an inverted light microscope, and we then confirmed that the isolate does not have visible attached creatures on the cell surface under a light microscope (magnification x1500) before the PCR amplification. Therefore it is thought that the bacterial 16S rDNA sequence from *B. bigelowii* is of *B. bigelowii* intracellular origin not a result of contamination. Our TEM studies showed that, in addition to common organelles, such as the nucleus, chloroplasts and mitochondria, *B. bigelowii* contains one or two spheroid bodies with internal lamellae (Figs. 2-3). Because other common organelles observed by the TEM cannot be the origin of the bacterial 16S rDNA sequences and no other additional structure, which can be the origin of the bacterial 16S rDNA, have been observed, it is highly likely that the UCYN-A 16S rRNA-like sequence came from the spheroid body of the *B. bigelowii*. Since we failed in amplification of *nif*H gene, we could not confirm that the spheroid body of *B. bigelowii* possesses the ability to fix nitrogen.

Zehr et al.[47] reported that UCYN-A lacks the genes for the oxygen-evolving photosystem II, RuBisCO, and the tricarboxylic acid cycle. Thompson et al.[20] discovered a symbiotic association between UCYN-A and a prymnesiophyte, and revealed that the prymnesiophyte received nitrogen from the cyanobacterium in exchange for transferring fixed carbon. The 18S rDNA sequence named BIOSOPE clone T60.34, which was obtained from the prymnesiophyte cell associated with UCYN-A, made a clade with Genotypes I-V of *B. bigelowii* and *C. parkeae* with high bootstrap values and posterior probability (94/1.00; ML/BI) (Fig. 4). Genotypes I-II, III, and IV-V were related to Intermediate form -A, Intermediate form -B, and Large form of *B. bigelowii*, respectively[13]. The size of the prymnesiophyte associated with the UCYN-A was slightly smaller than cells of the Small form of *B. bigelowii*, whose 18S rDNA sequence has not yet been determined. From these observations, Thompson et al.[20] suggested that the prymnesiophyte might represent an additional smaller size class of *B. bigelowii* that is adapted to the oligotrophic ocean. Presence or absence of calcareous pentaliths on the cell surface of the prymnesiophyte host was not confirmed by Thompson et al. [20]. We assume that their prymnesiophyte cell is related to, but different from *B. bigelowii* since it’s sequence BIOSOPE clone T60.34. falls separately at the base of the *B. bigelowii* clade in the 18S rDNA tree (Fig. 4), and they do not produce calcareous pentalith through its life cycle, since remains/fossils of the Braarudosphaeraceae with laminar structure do not occur from open ocean sediments. [43]

The manner of the association of UCYN-A with the prymnesiophyte partner [20] seems to be different from that of the spheroid body and of *B. bigelowii* (Intermediate form- B) reported here. Based on the sensitivity to disruption and observation by HISH-SIMS, Thompson et al. [20] assumed that the relationship between UCYN-A from the ALOHA station and the prymnesiophyte partner cell was extracellular. Krupke et al.[48] also reported extracellular relationships between the UCYN-A and unidentified eukaryotic cell. By contrast, the spheroid bodies of *B. bigelowii* Intermediate form-B (this study) were always observed inside the cells. Our results from TEM observation and molecular phylogenetic studies suggest that the presence of spheroid body of *B. bigelowii* sensu stricto (Intermediate form-B, 18S rDNA genotype III) is a result of endosymbiosis of the UCYN-A. The BIOSOPE clone T60.34. positions at the base of the *B. bigelowii* clade (Fig. 4). Therefore, it is thought that *B. bigelowii* acquired its UCYN-A endosymbiont after separation of the ancestor of *B. bigelowii* from the ancestor of the prymnesiophyte cell of Thompson et al.[20].

The 18S rDNA sequence of the MBIC 10518 culture strain, which corresponds to *Chrysochromulina brevifilum* MBIC 10518 of Thompson et al. [20], formed a clade with the sequences of *B. bigelowii* + *C. parkeae* + BIOSOPE T60.34 with high bootstrap supports and posterior probability (90/1.00; ML/BI), being located at the base of the clade. There is currently no published data concerning the MBIC 10518 strain except for the 18S rDNA sequence, and more detailed study of this strain (its morphology and life cycle) may also contribute to understanding the origin of the Braarudosphaeraceae.
